# The Emergence of an Urable Earth: How Early Planetary Evolution Shaped the Chemical Window for Life’s Origin

**DOI:** 10.3390/life16091436

**Published:** 2026-08-28

**Authors:** Meng Guo, Zekun Meng, Siyu Liu, Simon A. T. Redfern

**Affiliations:** 1 GAIA Lab, Department of Earth and Planetary Sciences, The University of Hong Kong, Hong Kong 999077, China; 2 Department of Physics, Hong Kong University of Science and Technology, Hong Kong 999077, China; 3 Asian School of the Environment, Nanyang Technological University, 50 Nanyang Avenue, Singapore 639798, Singapore; 4 Earth Observatory of Singapore (EOS), Nanyang Technological University, 50 Nanyang Avenue, Singapore 639798, Singapore

**Keywords:** planetary durability, earth system evolution, hadean earth environment, origin of life

## Abstract

Earth’s early history provides the only natural record for evaluating how planetary evolution can generate environments capable of initiating life. Here we review early Earth evolution through the lens of urability: the time-dependent capacity of planetary environments to support prebiotic chemistry progressing toward compartmentalized, self-propagating, information-bearing systems. We argue that urability is not a single globally habitable state, but a transient overlap among several coupled dimensions: liquid water availability, permissive temperature, ocean pH and salinity, access to bioessential elements, atmospheric shielding and volatile retention, and exposed or shallow environments that enable concentration, mineral catalysis, and wet–dry cycling. During the Hadean–early Archean transition, these dimensions were shaped by magma-ocean degassing, late accretion history, atmospheric compositional evolution from CO_2_-rich to more N_2_-dominated states, ferruginous ocean chemistry, tectonic recycling, continental growth, and intermittent land emergence. These processes created tradeoffs: high *p*CO_2_ may have enhanced abiotic nitrogen fixation but imposed hot and acidic conditions, whereas CO_2_ drawdown improved climate and ocean pH while weakening some fixed-nitrogen sources; ferruginous chemistry could locally enhance phosphate availability while also promoting nutrient scavenging; and tectonic recycling could stabilize the carbon cycle while generating chemically diverse but spatially intermittent land environments. We therefore frame life’s origin as a planetary timing problem, in which prebiotic opportunities opened and closed as multiple environmental constraints came into and out of overlap. This perspective motivates coupled models that resolve when and where water, temperature, pH, nutrients, energy, atmospheric photochemistry, and exposed land surfaces jointly produced urable environments on Earth and other rocky planets.

## 1. Introduction

Earth is the only confirmed planet known to host life and therefore provides the natural reference point for translating origin-of-life concepts into testable, time-evolving constraints. Geological and isotopic evidence suggests that life may have emerged early in Earth’s history, possibly within the Hadean–early Archean interval. Putative biosignatures have been reported from rocks as old as ~3.8 Ga [1], while more tentative claims based on Hadean zircon-hosted carbon inclusions extend the possible record to ~4.1 Ga [2]. Complementary constraints from comparative biology further suggest an ancient origin for the last universal common ancestor, although such inferences are indirect and do not constitute rock-based biosignatures [3]. If these lines of evidence are broadly correct, then by the late Hadean to early Archean period, Earth’s surface environment must have crossed a threshold from broadly hostile to chemically permissive. Candidate origin-of-life settings must therefore have sustained reaction networks for sufficiently long durations, and under suitable physicochemical conditions, to yield compartmentalized, self-propagating, information-bearing systems capable of Darwinian evolution [4,5,6,7].

While life today persists across a wide range of environments, this breadth does not imply that life’s origin was similarly unconstrained. Modern cells buffer external variability through membranes, selective transport, and homeostasis; before such systems evolved, prebiotic networks and protocell-like assemblies were likely more sensitive to physicochemical perturbations, and thus confined to comparatively restricted environments [8]. Nearly a century after the Oparin–Haldane chemical evolution framework was proposed, the hypothesized prebiotic pathways have expanded substantially and remain actively debated. A persistent challenge is not simply identifying plausible prebiotic reactions, but identifying pathways that remain viable under early Earth conditions that were spatially heterogeneous and temporally variable.

Despite diversity in proposed hypotheses, several requirements recur across frameworks [9,10], including (1) a reactive solvent, typically liquid water [11], (2) access to bioessential elements, at minimum carbon, hydrogen, nitrogen, oxygen, phosphorus, and sulfur (CHNOPS) [12,13], and (3) physicochemical conditions that permit reaction networks to persist and concentrate, including suitable ranges of temperature, ocean pH, and salinity, together with gradients or cycling that can drive accumulation and polymerization [14,15,16].

Crucially, these early Earth conditions were not static. Instead, they likely emerged through, and were repeatedly perturbed by, planetary evolution from the Hadean into the early Archean. Over the first billion years of Earth history, Moon formation, magma-ocean outgassing, solid Earth differentiation, late accretion, and the establishment of long-lived tectono-magmatic cycling could each substantially reconstruct surface conditions [17,18,19]. These processes governed not only whether liquid water could persist, but also how key elements were partitioned among terrestrial reservoirs and how rapidly permissive environments could form, evolve, and be erased. As a result, the plausibility of different prebiotic reaction networks was inherently time-dependent and set by coupled interior–surface evolution [20,21,22,23]. This motivates a central premise of this review: prebiotic pathways should be evaluated against explicit geological surface conditions produced by a time-evolving Earth system, which could repeatedly open and close windows of opportunity for prebiotic chemistry across the Hadean and early Archean.

To frame this time dependence of prebiotic pathways, we adopt the concept of urability [24], which focuses on the conditions required for life to begin rather than on long-term habitability alone. Urability is not a single universal recipe; instead, it is an overlap problem in which multiple geophysical and geochemical constraints must be collectively satisfied to create localized urable zones. Guided by this perspective, we emphasize a subset of evolving controls most directly tied to Hadean–Archean surface evolution and prebiotic chemical potential. We first evaluate when and how surface liquid water, and, by extension, stable oceans, could exist [25,26,27,28]. We then treat evolving surface temperature and ocean pH as first-order constraints on aqueous speciation and reaction viability, tracking their evolution through their shared sensitivity to atmospheric carbon dioxide levels (*p*CO_2_) [29,30,31]. We also examine how nutrient cycling in ferruginous oceans responds to the evolving chemical environment, including coupled tradeoffs between phosphorus availability versus fixed nitrogen inventories [32,33]. Finally, we consider land formation and tectonic mode, which regulate wet–dry cycling, reactive mineral surfaces, weathering fluxes, and long-term volatile and nutrient exchange between Earth’s interior and surface [34,35,36,37,38].

We synthesize these ideas into a unifying framework centered on a transient overlap time interval when atmospheric CO_2_ must decline sufficiently to yield clement climates and more favorable ocean pH, yet this same transition can reduce the production and persistence of oxidized nitrogen, while ferruginous ocean chemistry can simultaneously constrain phosphate availability. We argue that these coupled tradeoffs can generate a limited interval of elevated prebiotic chemical potential during the Hadean to early Archean transition, when late accretion and impact processing, volatile cycling, and ocean chemistry jointly governed the availability of key disequilibria and limiting reactants [29,31,39,40]. Our aim is therefore to reconstruct the evolving planetary boundary conditions within which such prebiotic pathways could have operated, rather than to review individual pathways of organic synthesis or to discriminate among specific origin-of-life scenarios.

## 2. Finding the Reactive Solvent: When Did the Hydrosphere Appear on Earth?

A stable hydrosphere is a prerequisite for the urability pathways emphasized here because it provides a long-lived solvent, enables transport and concentration of reactants, and supplies aqueous settings in which prebiotic chemistry can proceed [15,16]. This motivates a fundamental question: when and by what mechanisms did the first long-lived surface water reservoir, plausibly an ocean, appear on the Earth’s surface? Coupled interior–atmosphere models generally indicate that liquid oceans could have formed very early in the Hadean once the planet had cooled sufficiently for a steam atmosphere to condense, but they differ in regard to which processes dominated the timescale of water condensation, including radiative cooling and cloud effects, volatile partitioning and degassing, stellar irradiation, and impact reheating. Establishing when such an ocean emerged, however, first requires distinguishing the acquisition of Earth’s water inventory from its subsequent condensation at the surface.

The amount and provenance of water incorporated during planetary accretion cannot be inferred from the position of the snow line alone. In the protoplanetary disk, the snow line was a time-dependent physicochemical transition rather than a fixed boundary defined by a single condensation temperature. Its position varied with disk temperature, water-vapor pressure, opacity, accretional heating, stellar irradiation, and dust properties [41,42,43]. As the solar nebula evolved, radial drift of icy solids, vapor diffusion, and dynamical scattering redistributed water across this boundary and transported volatile-rich material into the terrestrial-planet-forming region [44,45,46]. The snow line therefore governed where water-bearing solids initially formed, but subsequent transport and accretion determined how much water was ultimately incorporated into Earth.

Available isotopic evidence indicates that Earth acquired much of its water from primitive asteroidal material with hydrogen- and nitrogen-isotopic compositions broadly resembling those of carbonaceous chondrites, rather than from the generally D-enriched comets measured to date [47]. A substantial fraction of this water was probably incorporated before the Moon-forming impact. Oxygen isotope constraints have been interpreted to suggest that Earth was already water-bearing at the time of lunar formation, although later accretion may have added a secondary fraction to the present inventory [48]. The Moon-forming impact should therefore be viewed primarily as an episode of volatile redistribution and partial loss, rather than as the event that separated an initially dry Earth from a subsequently wet one. During the impact, water would have been partitioned among molten silicates, vapor, ejecta, and the circumterrestrial disk. The critical distinction is between vaporization and irreversible loss: water converted into vapor was not necessarily removed from the Earth’s system unless it escaped gravitationally or was subsequently lost from the atmosphere [49]. Impact models indicate that giant collisions can erode substantial fractions of proto-atmospheres and that the presence of an ocean may enhance this erosion by increasing vapor production and altering shock propagation [50]. Nevertheless, such erosion need not have eliminated Earth’s entire pre-existing water inventory. Water that remained gravitationally bound—whether dissolved in the magma ocean or retained in the post-impact steam atmosphere—could subsequently have been redistributed during magma-ocean solidification and atmospheric cooling, ultimately condensing to form a surface ocean [19,51].

Late accretion after lunar formation likely modified an already substantial terrestrial water inventory rather than creating it from an initially dry state. Lunar isotopic and mass-balance models suggest that carbonaceous-chondrite-like asteroids were the principal carriers of water during this interval, whereas D-enriched comets supplied only a minor fraction, generally less than ~20% in the modeled mixtures [52]. Solar–wind implantation provides an additional but probably subordinate pathway. Laboratory experiments show that implantation of H^+^ into oxygen-bearing minerals on dust grains, meteoroids, and asteroids can generate and retain surface-bound OH and H_2_O, enabling nominally anhydrous material to carry small amounts of water [53]. However, the planetary significance of this mechanism remains uncertain because its production efficiency, survival during impact delivery, and integrated flux during accretion are poorly constrained. It is therefore best regarded as a supplementary contribution to late accretion, rather than as a primary source of Earth’s hydrosphere.

Whether inherited from the main accretionary phase or supplemented by late accretion, the surviving post-impact water inventory was likely distributed between the molten silicate interior and a H_2_O-rich atmosphere. The emergence of a stable surface hydrosphere therefore depended on the cooling of this coupled magma-ocean–atmosphere system. In the staged model of Abe [17], condensation occurred once net radiative cooling exceeded internal and impact-related heating, allowing atmospheric H_2_O to pass below its critical point and enter the liquid-water stability field. This can form a proto-ocean while leaving a dense residual atmosphere enriched in carbon species, predominantly CO_2_ and, under more reducing conditions, potentially CO. More recent coupled interior–atmosphere models with radiative transfer broadly support this picture but emphasize strong parameter dependence [51,54]. A thicker greenhouse atmosphere can substantially delay magma-ocean cooling; yet, for Earth-like irradiation and volatile (H_2_O and CO_2_) inventories, ocean condensation is often predicted on Myr timescales across parameter spaces (for example, ~1.5 Myr for Earth in [51]), with strong sensitivity to atmospheric opacity and the total volatile budget [51,54].

Complementary work emphasizes the supply side of the steam atmosphere. Volatile partitioning during magma-ocean crystallization can drive efficient late-stage degassing even for modest bulk water contents, building a steam atmosphere that collapses to liquid water once cooling permits [18]. A regime-based synthesis further clarifies why estimated ocean-formation times vary widely by distinguishing irradiation-controlled end-members [55]: Type I planets cool and solidify quickly enough that water vapor condenses soon thereafter, allowing an early, persistent surface ocean, whereas Type II planets sustain a long-lived magma ocean beneath a thick steam atmosphere for tens to hundreds of Myr, delaying condensation and potentially losing substantial water to space [55]. Earth is generally discussed as a Type I planet for plausible parameters.

**Figure 1:**
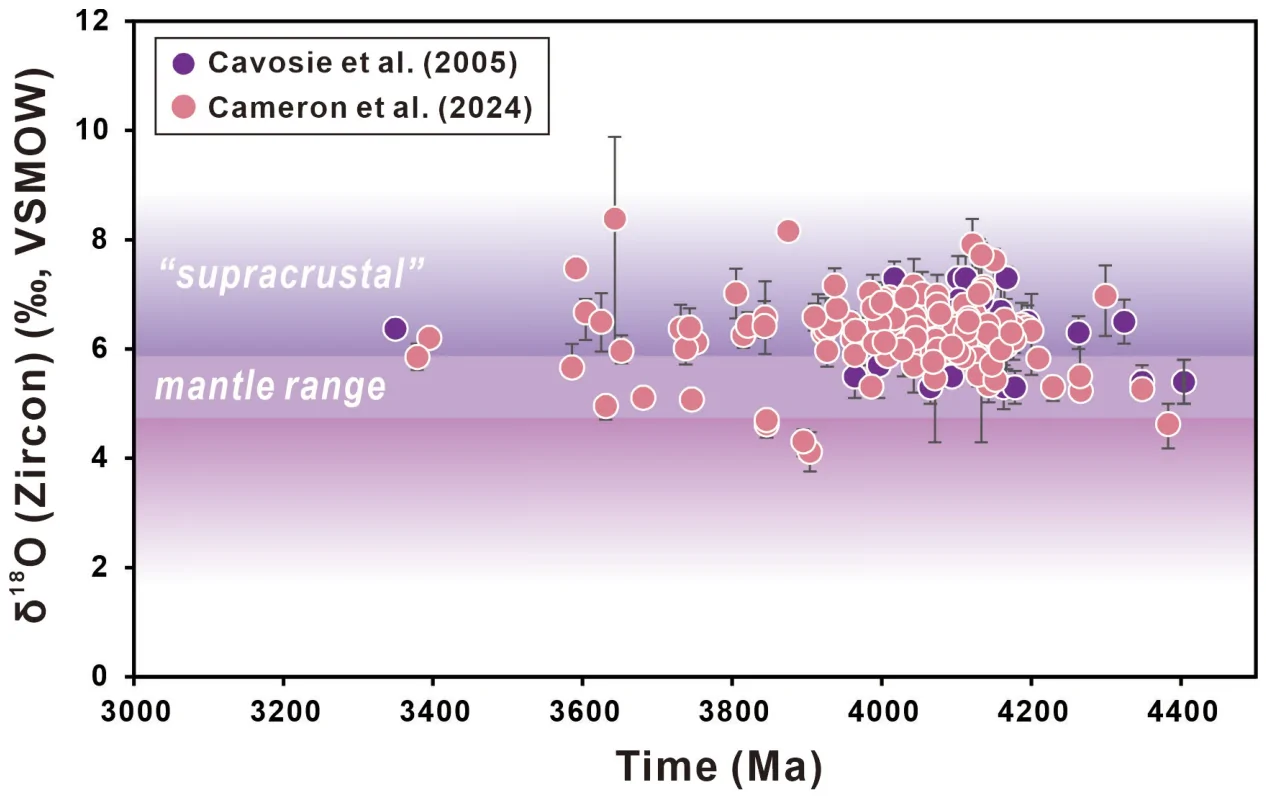
Modified from Cameron et al. [63] under CC BY 4.0. Oxygen isotope evidence for early crust–surface interactions recorded by Hadean–Eoarchean zircons [63]. Zircon δ^18^O values are plotted against crystallization age for dated zircon domains from Cavosie et al. [57] and Cameron et al. [63]. Data points represent domain-averaged δ^18^O values, with error bars indicating the within-domain δ^18^O range. The shaded bands mark the reference fields for mantle-like and supracrustal zircon δ^18^O values. The supracrustal field is characterized by elevated δ^18^O relative to the mantle-like field, reflecting zircon crystallization from magmas that incorporated materials altered by low-temperature surface fluids.

Proxy constraints provide an independent observational basis for assessing the presence of Hadean surface liquid water and, by extension, the plausibility of Hadean oceans [25,26,27,28]. Because essentially no intact Hadean seafloor is preserved, some of the strongest constraints come from detrital zircons, especially those from the Jack Hills, which record the compositions and histories of their source rocks. Many Hadean zircons exhibit elevated δ^18^O values relative to mantle compositions, and although alternative interpretations exist for the provenance and processing of the altered component, these signatures are widely discussed as evidence for incorporation of supracrustal material that experienced low-temperature water–rock interactions near the surface prior to burial, metamorphism, and remelting [26,56,57,58,59] (Figure 1). Anchored by U-Pb ages extending back to ~4.4 Ga [25], these observations imply that by the mid-Hadean, Earth hosted near-surface environments where liquid water interacted with crustal materials, consistent with an early hydrosphere and substantial surface water reservoirs. Additional zircon-based constraints are often discussed in the same context. The Ti-in-zircon thermometry indicates that many Hadean zircons crystallized at temperatures comparable to modern felsic magmatism, consistent with generation from evolved crustal sources [27]. Mineral inclusions and inclusion-thermobarometry in ~4.0 Ga zircons further point to differentiated, moderate-pressure crustal environments early in Earth history, supporting the view that evolved crust and surface-interacting reservoirs existed during the Hadean [60,61]. Together, these zircon-based proxies converge on the inference that liquid water was present at or near Earth’s surface very early, and that ocean formation by ~4.4–4.3 Ga is consistent with the available mineral record. As with any indirect archive, these proxies are best interpreted as constraints on the existence of surface liquid water and water–rock interactions, rather than as uniquely specifying the global extent or uninterrupted stability of oceans through the entire Hadean. Nonetheless, they provide some of the most direct observational windows currently available on early Earth’s hydrosphere [28,62].

Large impacts can transiently vaporize surface oceans and generate short-lived, high-temperature atmospheres, but once silicate vapor and condensates rain out, the remaining steam atmosphere can cool under an outgoing longwave radiation limit and recondense liquid water on short timescales, compared with geologic intervals, allowing oceans to be re-established even after severe perturbations [64]. Importantly, impact heating need not imply global sterilization. Thermal models suggest that subsurface refugia can persist through bombardment and that impacts can generate long-lived hydrothermal circulation, maintaining aqueous environments even when the surface is episodically disrupted [65,66]. Because the bombardment chronology remains debated, the frequency and severity of these reset events are uncertain. Several analyses favor a prolonged decline in impact flux rather than a single narrow cataclysm, implying that impact-driven perturbations may have been distributed across hundreds of millions of years [67,68].

These considerations frame the key observations and uncertainties in reconstructing the initial ocean conditions relevant to early surface chemistry. A conservative synthesis is therefore that Earth’s earliest hydrosphere emerged through a sequence rather than a single event. Water was acquired during planetary accretion from dynamically mixed, volatile-bearing materials, a substantial fraction may have survived or been redistributed during the Moon-forming impact, and additional water was supplied during subsequent late accretion. Magma-ocean degassing and radiative cooling then allowed the retained inventory to condense into surface oceans, which were repeatedly disturbed but not necessarily permanently removed by later bombardment. By at least ∼4.4 Ga, zircon evidence indicates that liquid water was interacting with crustal materials, although neither the global extent nor the uninterrupted continuity of these early oceans can be uniquely established.

## 3. Atmospheric Compositional Evolution: From CO_2_-Dominated to N_2_-Dominated

### 3.1. Early pCO_2_ in the Hadean and Archean: Magma-Ocean Degassing and Geological Constraints

The compositional evolution of an atmosphere constitutes a first-order reorganization of a planet’s surface environment because it simultaneously modulates the climate, sets ocean chemistry through dissolved inorganic carbon, and governs photochemistry and atmospheric escape that together shape redox budgets and long-term volatile retention [23,69,70,71]. Within the Solar System, Earth appears unique in having transitioned from an early CO_2_-dominated atmosphere toward a long-lived N_2_-dominated condition and an altered photochemical regime. In contrast, Venus retained a massive CO_2_ atmosphere associated with extreme greenhouse effects, whereas Mars evolved toward a thin, CO_2_-dominated one shaped by atmospheric loss and surface sequestration, with major consequences for climate stability [69,72,73]. This contrast motivates treating atmospheric compositional evolution not as background context, but as a controlling geological condition for time-resolved assessment of prebiotic chemical opportunity.

On Earth, post-magma-ocean solidification models consistently imply that the earliest atmosphere could have been extremely carbon-rich. Depending on volatile inventories, magma-ocean redox state, and the stage considered (e.g., near the end of crystallization or after H_2_O condensation), estimated initial atmospheric *p*CO_2_ commonly spans tens to a few hundred bars [17,18,31,64,74]. At magma-ocean temperatures, carbonate minerals are unstable, favoring extensive outgassing during and after crystallization and leaving much of the carbon in the melt–vapor system as C–O species rather than as solid carbonates [17,75]. Classic degassing calculations therefore tend to yield proto-atmospheres with 10^2^ bar CO_2_ accompanied by a massive steam component prior to condensation, implying strong greenhouse forcing under the faint young Sun [18,74].

Models that explicitly treat magma–gas redox equilibria and volatile speciation refine this picture by showing that the very earliest atmosphere may be more reduced, with appreciable CO and H_2_ alongside H_2_O, and that subsequent cooling, steam collapse, and hydrogen escape can drive oxidation and a shift toward CO_2_ dominance under carbon-rich conditions [75,76]. Consistent with these end-member initial conditions, steam-collapse scenarios propose that once the steam atmosphere collapses, a liquid-water ocean at ~500 K can coexist with ~10^2^ bar CO_2_, with the persistence of this warm state controlled by the efficiency and timescale of CO_2_ drawdown [64,77,78]. More recently, coupled degassing–geodynamics models similarly suggest that a large fraction of Earth’s carbon can initially partition into the atmosphere, allowing post-solidification *p*CO_2_ to approach ~200 bar before progressive carbon sequestration into the crust and mantle over time [31]. Despite methodological differences, these studies converge on the conclusion that early Earth’s atmosphere contained orders of magnitude more CO_2_ than today (for comparison, modern *p*CO_2_ is ~10^−4^ bar). Climate calculations further indicate that very high CO_2_ abundances can sustain extremely warm post-condensation states; however, conclusions about whether CO_2_ alone can induce a classical runaway greenhouse depend on model assumptions (e.g., one-dimensional radiative-convective structure, H_2_O profiles, and treatment of clouds), and one-dimensional radiative-convective calculations suggest no runaway for *p*CO_2_ up to at least ~100 bar under typical setups [79].

**Figure 2:**
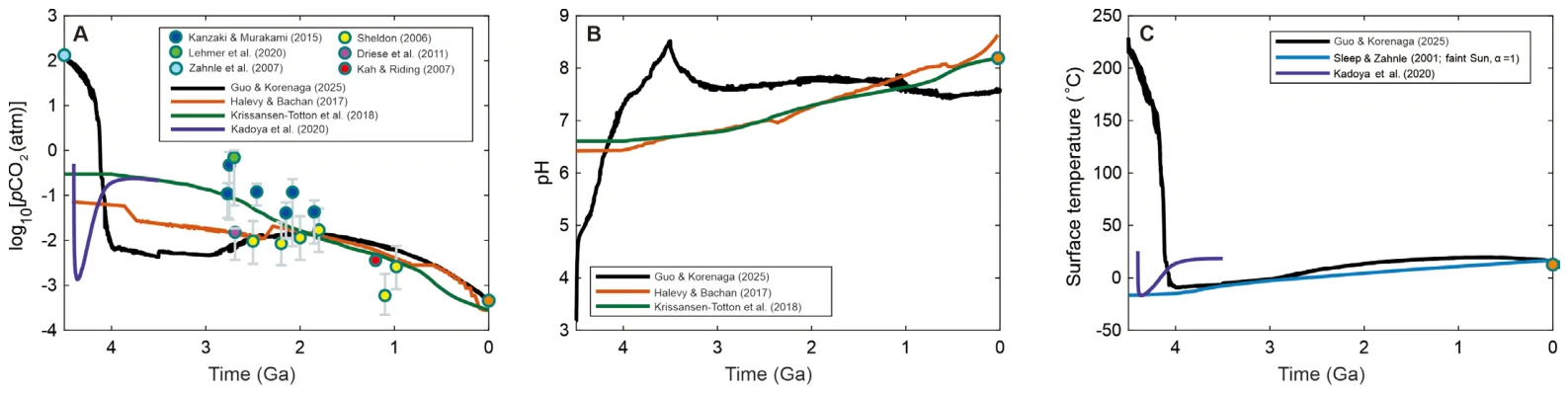
Modelled long-term evolution of Earth’s surface conditions, modified using the model results of Guo and Korenaga [37]. The black lines represent the median Monte Carlo results of Guo and Korenaga [37], and the orange dots are the present-day Earth surface conditions. (**A**) Atmospheric partial pressure of CO_2_ (in log_10_ scale), compared with proxy data from Sheldon [81], Driese et al. [82], Kanzaki and Murakami [83], Kah and Riding [91], Lehmer et al. [92], and Zahnle et al. [64]. Published model estimates from Halevy and Bachan [30], Krissansen-Totton et al. [93], and Kadoya et al. [94] are shown in orange, green, and purple, respectively. (**B**) Ocean pH, compared with the median model solutions of Halevy and Bachan [30] and Krissansen-Totton et al. [93], shown in orange and green, respectively. (**C**) Surface temperature through Earth history, compared with results from Sleep and Zahnle [21] and Kadoya et al. [94], shown in blue and purple, respectively.

On the other hand, independent observational constraints from the Archean indicate that this initially CO_2_-enriched atmosphere did not persist long into Earth history. Paleosol-based reconstructions generally suggest that by the Neoarchean to Paleoproterozoic periods, atmospheric *p*CO_2_ had fallen well below estimated Hadean values, although inferred ranges remain method-dependent and sensitive to assumptions about soil formation, temperature, and pore-fluid chemistry [80,81,82,83] (Figure 2). Mechanistically, such a decline is expected once a CO_2_-rich atmosphere coexists with surface liquid water and a reactive silicate crust: CO_2_ dissolves into the ocean and is consumed through continental weathering (where land is available) and alteration of the basaltic seafloor, generating alkalinity and promoting carbonate formation that can be buried and recycled into the interior.

Taken together, the model and proxy constraints discussed above are intended to define the dominant, long-lived atmospheric background rather than the transient or spatially localized chemical states accessible on early Earth. Large impacts and associated metal–water reactions could have generated short-lived H_2_-rich atmospheres, while shock chemistry, lightning, ultraviolet irradiation, and mineral–surface reactions may have produced localized enrichments in CO, CH_4_, HCN, and other reduced or prebiotically relevant species [84,85,86,87]. Such environments could have been important for localized prebiotic synthesis, but their integrated source strengths, lifetimes, spatial extents, and recurrence frequencies remain poorly constrained. They have also not been shown to dominate the bulk atmospheric carbon inventory over the long timescales considered here. We therefore treat these reduced chemical states as transient perturbations superimposed on a predominantly CO_2_-rich global background, rather than as alternative long-lived atmospheric end-members.

### 3.2. Mechanisms and Timescales of CO_2_ Drawdown

Exactly how long hot post-condensation, high-CO_2_ climates persisted remains debated because the controlling timescales depend on the efficiency of CO_2_ sinks, the vigor of crustal production and recycling, and the degree to which impacts intermittently reset surface conditions.

Post-impact models argue that drawdown from extreme end-member states could be rapid on geological timescales, yet still span orders of magnitude depending on assumptions [64,78]. Carbon cycling simulations emphasize that vigorous hydrothermal circulation and seafloor weathering under high heat flow can make CO_2_ drawdown efficient when crustal production is rapid [21], consistent with evidence for extensive hydrothermal alteration of the Archean oceanic crust [88]. Coupled geochemical and geodynamic models further quantify substantial carbon uptake in the upper oceanic crust and highlight recycling as an efficient pathway for long-term storage and exchange with the mantle [31].

Additional transient sinks have been proposed in which impact-ejecta weathering enhances alkalinity generation and consumes atmospheric CO_2_ during heavy bombardment [89,90]. The net effectiveness of this pathway, however, depends on how much carbon stored in altered ejecta and crust is later returned by metamorphism and recycling, particularly if impacts are frequent enough to repeatedly heat and rework near-surface reservoirs.

Most recently, Guo and Korenaga [37] coupled a global carbon cycle to marine charge balance with time-dependent interior evolution, and proposed that enhanced silicate weathering on both the oceanic and continental crusts in the Hadean could have driven CO_2_ drawdown consistent in magnitude with the gap between initial *p*CO_2_ estimates and Archean proxy bounds (Figure 2A). Once CO_2_ is substantially reduced, a dynamic balance among sources (degassing and metamorphic return) and sinks (continental and seafloor weathering) would maintain a more modest CO_2_ level in the coupled atmosphere–ocean system.

### 3.3. Transition to an N_2_-Dominated Atmosphere

As CO_2_ is progressively removed by water–rock reactions and carbon burial, the atmosphere can evolve from a CO_2_-dominated state toward an increasingly N_2_-dominated regime because CO_2_ has efficient low-temperature sinks, whereas N_2_ generally lacks equally fast abiotic pathways for net removal from the atmosphere over comparable timescales. In the modern Earth system, the creation of reactive nitrogen is dominated by biology and, in the Anthropocene, by human activities, which is commonly summarized as ~413 Tg N yr^−1^; by contrast, lightning produces only a few Tg N yr^−1^ of NO_x_, implying that current abiotic nitrogen fixation contributes on the order of ~1% of the modern fixed-N budget [95,96,97].

Early Earth conditions likely differed in ways that enhanced abiotic N_2_ fixation. First, experiments and modeling indicate that lightning-induced fixation can be far more efficient in CO_2_-rich atmospheres than today, with enhancements spanning orders of magnitude across plausible atmospheric compositions and historic *p*CO_2_ levels [32,98]. Second, impacts provide an additional episodic source through energy-driven fixation of atmospheric N_2_, commonly parameterized as a declining flux through time as bombardment wanes [99,100]. However, whether these enhanced sources translate into substantial long-term atmospheric N_2_ drawdown depends on the efficiency of net sinks for fixed nitrogen. Sequestration occurs through adsorption, burial, and incorporation of reduced nitrogen in sediments and altered crust, but these sinks are balanced by return fluxes from metamorphism and mantle degassing, with partitioning and speciation sensitive to redox state and fluid–melt chemistry [101,102].

To quantify these couplings in a time-evolving Hadean physicochemical environment, Guo and Korenaga (in revision) link nitrogen cycling to the evolving carbon cycle by allowing lightning fixation to vary with *p*CO_2_ and incorporating impact fixation. Even with elevated abiotic fixation under CO_2_-rich Hadean conditions, their results suggest that atmospheric *p*N_2_ could remain high throughout the prebiotic interval, declining only to roughly five times its present-day level (PAL) through abiotic pathways alone [33]. Together, rapid CO_2_ drawdown combined with comparatively inefficient net N_2_ removal provides a physically motivated basis for a shift from CO_2_-dense to N_2_-dominated atmospheres, with downstream consequences for prebiotic chemistry through its effects on the Earth’s surface temperature, ocean pH, and upper-atmosphere photochemistry and escape.

## 4. Shaping Prebiotic Environments: Evolving Surface Temperature, Ocean Chemistry, Nutrient Availability, and Photochemical Regimes

### 4.1. The pCO_2_-Driven Early Climate Trajectories

Atmospheric compositional evolution reshapes Earth’s surface boundary conditions in multiple coupled ways. Consistent with the urability framework introduced above, we treat prebiotic opportunity as an overlap problem in which localized urable zones exist only where multiple constraints are simultaneously satisfied. Here, we focus on how *p*CO_2_-driven changes regulate surface temperature and ocean pH, which in turn govern aqueous speciation, reaction kinetics, the stability of key intermediates, nutrient availability, and the persistence of protocell-like compartments. We also discuss how the changing of atmospheric composition influences the broader photochemical environment and upper-atmosphere escape, with downstream consequences for redox evolution and long-term volatile retention.

For prebiotic chemistry, the key implication of reconstructing surface climate evolution is not to identify a single “target” global temperature, but to evaluate whether surface and near-surface environments increasingly overlapped with permissive thermal ranges for synthesis, stability, and accumulation. As heuristic bounds informed by biology, the lower end of permissive conditions is often anchored by the ability of microbes to reproduce under subfreezing conditions (commonly cited near −15 °C), whereas the upper end is set by the highest confirmed growth temperatures (near ~122 °C), above which biomolecules and cellular systems incur rapid, irreversible damage [103,104]. From a prebiotic perspective, the same qualitative tradeoff applies: elevated temperatures can accelerate reaction rates, but hot aqueous environments also promote hydrolysis and decomposition of sugars, nucleobases, and polymers, reducing the likelihood of sustained accumulation of many key organics as temperatures approach and exceed the hyperthermophile limit [11,105].

Following magma-ocean solidification, a CO_2_-dominated atmosphere can sustain extremely warm post-condensation states, with surface temperatures potentially exceeding ~200 °C in the hottest end-members [17,64]. In steam-atmosphere collapse scenarios, such very warm oceans may persist for ~10–100 Myr, with the duration governed primarily by the efficiency and timescale of CO_2_ drawdown [64,78]. As CO_2_ is progressively removed by weathering and crustal alteration, Earth can transition toward more temperate regimes. In some scenarios, CO_2_ drawdown combined with the faint young Sun may yield intermittently cold climates, depending on the balance between weathering sinks, metamorphic and degassing sources, and other greenhouse contributors [21,64,89,93,106]. Taken together, these model results imply that the most extreme post-condensation thermal states were likely transient, while more clement conditions became increasingly widespread and persistent from the middle to late Hadean, expanding the spatial and temporal extent of environments compatible with sustained prebiotic chemistry.

### 4.2. The pCO_2_-Driven Ocean pH Evolution

In parallel with thermal evolution, the pH of Earth’s aqueous environments is also expected to shift in response to declining atmospheric *p*CO_2_. In general, high atmospheric *p*CO_2_ elevates dissolved inorganic carbon and proton activity, tending to acidify seawater, whereas declining *p*CO_2_, coupled with sustained water–rock exchange, drives waters toward more neutral to alkaline conditions [30,37,93]. Because the pH of oceans and inland water environments exert first-order control on aqueous speciation, metal solubility, mineral buffering, and reaction kinetics, this *p*CO_2_-controlled pH trajectory acts as a time-dependent filter on prebiotic plausibility by shaping both the stability of organic intermediates and the availability of key ions and nutrients. Strongly acidic conditions can accelerate hydrolysis of labile organics and destabilize amphiphile assemblies, while highly alkaline conditions impose different constraints through speciation shifts and altered phosphate or metal availability. Consequently, many proposed prebiotic scenarios are favored within a mildly acidic to mildly alkaline window, with the operative bounds depending on the pathway and environmental setting [9,15,16].

Compared with earlier ocean-pH reconstructions [30,93], Guo and Korenaga [37] present a coupled global carbon-cycle and marine-chemistry framework explicitly initialized with the high post-magma-ocean pCO_2_ conditions implied by degassing models. In their calculations, rapid CO_2_ drawdown during the Hadean, driven by elevated weathering enabled by vigorous crustal production and recycling, yields a concrete trajectory for the timing and magnitude of ocean neutralization: seawater evolves from strongly acidic end-member conditions (pH ~5 under high *p*CO_2_) toward near-neutral values (pH ~6–7) by approximately 4.0 Ga [37]. As the early oceans were likely anoxic and ferruginous, the *p*CO_2_-driven rise in pH does not only neutralize the carbonate system; it also reorganizes Fe^2+^ solubility and Fe mineral speciation, thereby controlling the strength of iron-mediated sinks for phosphate and oxidized nitrogen and setting the nutrient landscape relevant to prebiotic chemistry.

### 4.3. Nutrient Availability in Early Ferruginous Oceans Under pCO_2_-Driven pH and Temperature Evolution

**Figure 3:**
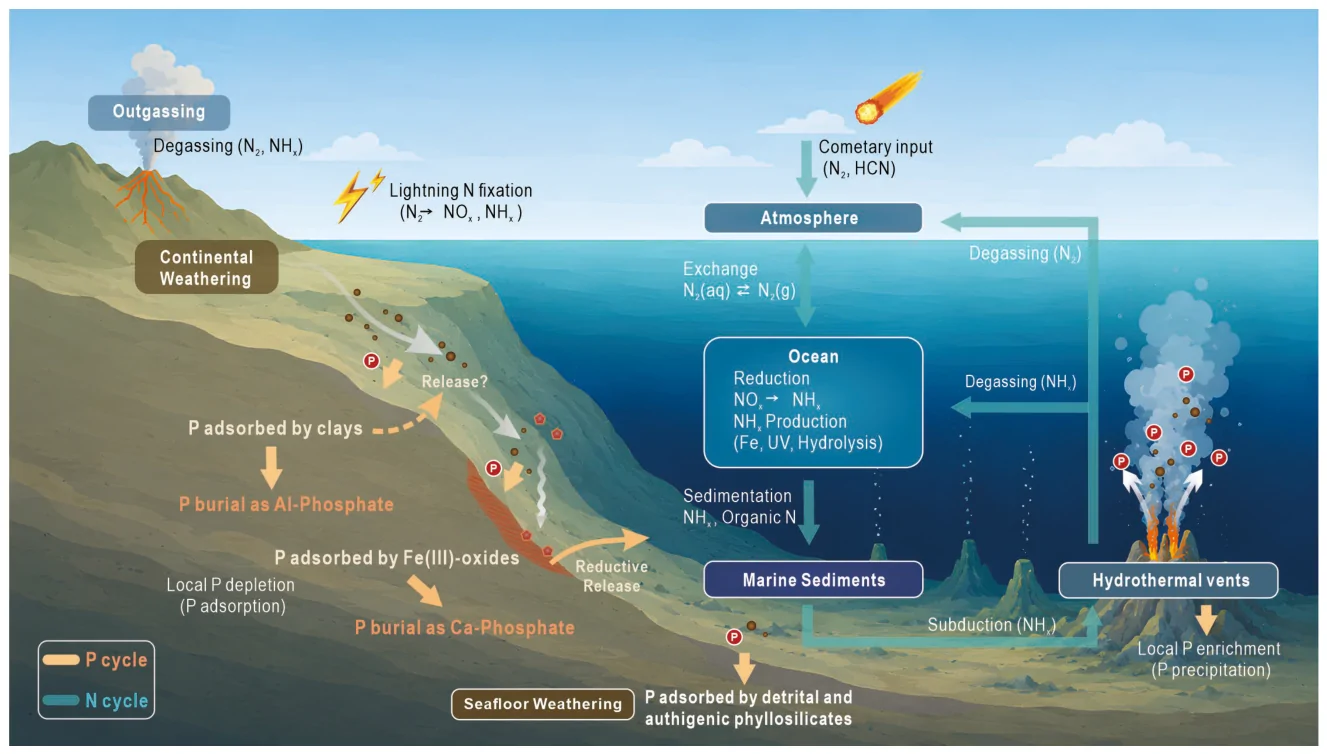
Schematic illustration of biogeochemical cycling and exchange pathways of nitrogen and phosphorus among the surface reservoirs of the early Earth. Yellow arrows denote phosphorus transfer processes, blue arrows denote nitrogen transfer processes, and white arrows denote continental weathering inputs.

Nutrient availability in Hadean–Archean ferruginous oceans was shaped by the evolving physicochemical state of the Earth’s surface, rather than by nutrient supply alone. Atmospheric *p*CO_2_, seawater pH and temperature, hydrothermal circulation, water–rock reaction intensity, and marine redox structure jointly regulated the delivery, speciation, and removal of bioessential elements [107]. High-*p*CO_2_ conditions likely favored warmer and more acidic to near-neutral oceans, enhancing solute mobilization from mafic crust and hydrothermal systems. Subsequent changes in *p*CO_2_ and seawater pH would have altered the stability of Fe-bearing minerals, phosphate phases, carbonate systems, and reactive particle surfaces [30,33,93,108]. Within this framework, Fe acted not only as a micronutrient but also as a major geochemical regulator of nutrient availability. By shifting among dissolved Fe(II), authigenic Fe(II)-silicates and carbonates, photochemically produced Fe(III)-bearing particles, and reactive mineral surfaces, Fe could control whether nutrients remained in solution, were stabilized by aqueous complexation, scavenged onto particles, or transferred into sediments [109,110,111,112]. Early nutrient availability should therefore be viewed as a dynamic balance among geological supply, aqueous stabilization, mineral precipitation, particle scavenging, and burial (Figure 3). Phosphorus and nitrogen were embedded in this shared ferruginous framework, but their dominant controls differed: phosphorus was governed mainly by mineral–water equilibria and Fe-mediated surface reactions, whereas oxidized nitrogen availability depended more directly on redox transformations and the emergence of biological nitrogen cycling.

**Phosphorus.** The central issue in the Hadean–Archean phosphorus cycle is not simply whether phosphorus can be delivered to the ocean, but whether it could remain bioavailable after entering a ferruginous, silica-rich, and mineral-reactive marine system. Abiotic transfer pathways, including hydrothermal circulation, crustal recycling, seafloor alteration, sedimentation, and subduction, could have redistributed phosphorus among solid Earth, oceanic, and sedimentary reservoirs before the emergence of a strongly biologically modified P cycle [113,114]. However, delivery alone does not determine ecological availability. Once supplied to surface or hydrothermal marine environments, phosphate can either persist in dissolved form, become stabilized by aqueous complexes, adsorb onto reactive mineral surfaces, precipitate as authigenic phases, or be buried in sediments. The early P cycle was therefore controlled by the competition between input, aqueous retention, particle reactivity, and burial.

Ferruginous seawater may have created local windows of enhanced phosphorus availability. Traditional models commonly assumed that phosphate was scarce on the prebiotic Earth because apatite-group minerals are poorly soluble and because Fe^2+^ under anoxic conditions could limit dissolved phosphate concentrations through mineral saturation or precipitation [109,115,116]. More recent experimental and thermodynamic work, however, suggests that Fe^2+^ could also increase the apparent solubility of phosphate minerals by forming aqueous Fe^2+^–phosphate complexes under weakly acidic to near-neutral conditions [111]. At the solution-chemistry level, this effect reflects a decrease in the activity of free phosphate species rather than a simple increase in total phosphorus alone: as phosphate becomes progressively deprotonated with increasing pH, Fe^2+^ can associate with phosphate oxyanions as aqueous ion pairs or complexes, allowing more total dissolved P to be maintained before Fe- or Ca-phosphate saturation is reached [117,118,119]. In vent-proximal systems, this dissolved Fe–P reservoir could then be converted into nanoparticulate mineral P during hydrothermal fluid–seawater mixing, where changes in pH, temperature, Ca, F, and phosphate activity promoted fluorapatite saturation together with Fe(II)-silicate precipitation [120]. Such evidence does not require the entire ocean to have been phosphate-rich, but it does imply that hydrothermal plumes, vent-proximal basins, and chemically reactive seafloor environments could have generated localized nutrient-rich settings.

Yet the same ferruginous and mineral-reactive conditions that promoted phosphate retention in solution could also accelerate its removal from seawater. One important pathway involved Fe^2+^-mediated adsorption of phosphate onto phyllosilicates. Under anoxic, Fe-rich conditions, Fe^2+^ can act as a bridging cation between negatively charged phosphate species and negatively charged mineral surfaces, thereby enhancing phosphate adsorption onto clay minerals and Fe–Mg silicates [112]. Mechanistically, Fe^2+^ does not simply remove phosphate by precipitation; instead, it can form ternary surface complexes in which phosphate oxygens coordinate Fe^2+^ that is itself associated with deprotonated hydroxyl or permanent negative-charge sites on phyllosilicates [121,122]. This mineral-scale bridge neutralizes electrostatic repulsion between phosphate and negatively charged clay or Fe–Mg silicate surfaces, thereby converting otherwise weakly reactive phyllosilicate surfaces into efficient phosphate-retention sites [123,124,125]. This process would have been relevant both in shallow settings influenced by continental weathering and in submarine environments shaped by alterations to mafic and ultramafic crusts. Consequently, under the Hadean scenario of declining *p*CO_2_ and progressively increasing oceanic pH [37], such Fe–P coupling mechanisms may have played an increasingly important role. In parallel, redox-driven particle formation may have provided another efficient removal pathway. Photochemical oxidation of Fe(II), whose efficiency depends on pH and light wavelength, could have generated reactive Fe(III)-oxide particles capable of scavenging phosphate from seawater [110]. In early O_2_- and O_3_-poor atmospheres, enhanced penetration of ultraviolet radiation may have further favored such photochemical reactions. Thus, iron likely played a dual role in the early P cycle: it could stabilize phosphate in solution through aqueous complexation, but it could also promote phosphate capture through mineral adsorption and oxide scavenging.

The Hadean–Archean ocean was therefore unlikely to have been uniformly P-rich or uniformly P-poor. Instead, phosphorus availability was probably spatially and temporally heterogeneous. Hydrothermal systems, vent-proximal basins, and localized reaction zones may have sustained transiently high phosphate activity, providing favorable environments for prebiotic chemistry and early microbial metabolism. At the same time, widespread adsorption onto phyllosilicates, authigenic mineral formation, Fe(III)-oxide scavenging, and sedimentary burial would have limited the persistence and spatial expansion of dissolved bioavailable P. This balance between local enrichment and efficient mineral capture may explain how early oceans could generate nutrient-rich niches while maintaining low average phosphate availability at the global scale.

**Oxidized nitrogen.** A major abiotic source of oxidized nitrogen on early Earth was atmospheric NO_x_ production by lightning, whose efficiency was sensitive to atmospheric composition and may have been enhanced under more CO_2_-rich conditions relative to more N_2_-dominated states [32,98,126]. However, atmospheric production alone would not have guaranteed a persistent oceanic nitrate–nitrite reservoir. Once NO_3_^−^ and NO_2_^−^ entered an anoxic, Fe^2+^-rich ocean, they would have remained soluble but redox-labile. Fe^2+^-coupled reactions in the water column and on mineral surfaces could reduce nitrate and nitrite toward reduced or gaseous nitrogen products, including NH_4_^+^, N_2_O, and N_2_, depending on reaction pathway, mineral catalysis, pH, and temperature [33,127,128,129]. Hydrothermal circulation would have provided an additional sink for oxidized nitrogen, favoring reduced-N products under relevant temperature and pH conditions [130,131,132]. Before the establishment of biological nitrification, persistent nitrate–nitrite inventories would therefore have depended on whether abiotic atmospheric production could outpace reductive destruction in ferruginous seawater and hydrothermal systems.

This distinction highlights an important asymmetry between phosphorus and oxidized nitrogen in early ferruginous oceans. Phosphorus availability was strongly shaped by mineral solubility, aqueous complexation, surface adsorption, and particle scavenging, whereas nitrate and nitrite were limited mainly by redox instability after delivery to the ocean. This does not imply nitrogen starvation in a total sense, because reduced nitrogen species such as NH_4_^+^ may have been more persistent under anoxic conditions. Rather, it suggests that oxidized nitrogen was unlikely to accumulate as a stable oceanic reservoir in the absence of sustained production and biological recycling. From an origin-of-life perspective, ferruginous, low-sulfate Hadean–Archean oceans may therefore have allowed local phosphate enrichment while simultaneously making persistent oxidized-N inventories difficult to maintain. Coupled with long-term changes in atmospheric *p*CO_2_, seawater pH, and temperature [37], these redox–nutrient linkages would have constrained the physicochemical window in which phosphate availability, nitrogen speciation, and broader environmental conditions overlapped to support prebiotically favorable or early habitable aqueous environments.

### 4.4. Atmospheric Composition Controls on Upper-Atmosphere Escape and Surface Photochemical Environments

Beyond lower-atmosphere climate and ocean chemistry, atmospheric composition also regulates upper-atmosphere structure, atmospheric escape, volatile retention, and planetary redox evolution [23,133]. Escape affects redox evolution primarily through net hydrogen loss: when hydrogen derived from H_2_O, H_2_, CH_4_, or other reduced species escapes to space, reducing power is removed and the residual planet is oxidized [134,135,136].

For Earth, long-term ocean retention depended critically on the maintenance of an effective atmospheric cold trap. Under temperate conditions, condensation near the tropopause limits the upward transport of water vapor, keeping the stratosphere relatively dry and thereby restricting photolysis-driven hydrogen escape [137,138]. If, however, higher orders of magnitude of *p*CO_2_ after magma-ocean solidification drove surface temperatures sufficiently high, the cold trap could have weakened or failed, producing a moist upper atmosphere in which H_2_O was more readily photolyzed and hydrogen escape became more efficient [137,138,139]. In this sense, rapid CO_2_ drawdown may have been important not only for cooling the surface and moderating ocean pH, but also for helping preserve Earth’s water inventory by maintaining a relatively dry stratosphere.

The effect of CO_2_ on atmospheric escape is nevertheless complex. Although CO_2_-rich atmospheres can warm the lower atmosphere and, under sufficiently hot conditions, weaken the cold trap, CO_2_ and related carbon-bearing species can also cool the middle and upper atmosphere radiatively, thereby reducing thermospheric expansion and potentially suppressing hydrodynamic escape [71,139,140]. Thus, in the Hadean, whether CO_2_-rich states enhanced or limited hydrogen escape depended on the abundance of H-bearing species, the efficiency of the cold trap, the thermal structure of the upper atmosphere, and the high early EUV flux. CO_2_-rich conditions may therefore have moderated, but did not necessarily eliminate, oxidation driven by hydrogen escape [23,135].

As CO_2_ was progressively sequestered and N_2_ became more prominent, changes in mean molecular weight, radiative cooling efficiency, and upper-atmospheric structure may have altered the efficiency of different escape channels. However, the net effect on atmospheric loss would have depended on the decline of solar EUV flux through time, the abundance of residual radiative coolants, atmospheric redox state, and magnetic or solar–wind interactions [71,133,139,140]. This framing highlights how broadly similar terrestrial planets can diverge in atmospheric pressure, composition, ocean retention, and redox trajectory, with CO_2_/N_2_ ratios acting as one important control parameter alongside volatile inventories, stellar forcing, impact history, and surface–interior sinks [23,141].

Atmospheric composition also shaped the photochemical environment relevant to prebiotic synthesis. As discussed above, the production rate of fixed nitrogen as NO_x_ by lightning is sensitive to the CO_2_/N_2_ ratio because CO_2_-bearing gas mixtures alter high-temperature reaction pathways and post-discharge chemistry that regulate NO_x_ yields [32,98,127]. Radiative-transfer models further show that plausible CO_2_–N_2_ mixtures generate distinct surface UV regimes, which could either enable or suppress UV-driven prebiotic reaction networks depending on wavelength-dependent shielding and photochemical context [70,142,143]. A physically consistent account of atmospheric evolution therefore requires coupling lower-atmosphere climate and carbonate chemistry, largely governed by *p*CO_2_, with upper-atmosphere structure and escape, which are sensitive to *p*CO_2_/*p*N_2_, H-bearing species, and EUV forcing. Such coupling is needed to define the time-dependent environmental matrix within which prebiotic chemical networks could emerge and persist.

## 5. Hadean Tectonic Regime and Continental Evolution as Drivers of Urability

A central link between Earth’s tectonics regime and prebiotic chemistry is that early Earth likely emerged from magma-ocean solidification with an atmosphere containing very high *p*CO_2_. This excess CO_2_ had to be removed efficiently to generate surface conditions compatible with liquid-water stability, a moderate ocean pH, a suitable surface temperature range, a functioning atmospheric cold trap, and chemically persistent prebiotic environments. Near-surface basalt carbonation could have provided an important early sink, especially under vigorous hydrothermal circulation, but a simple capacity estimate demonstrates why this process alone is insufficient.

Many estimates of carbon uptake into oceanic crust emphasize alteration depths of a few hundred meters, with much of the carbonate inventory concentrated in the upper ~300 m to 500 m of the basaltic crust [88,144]. Once this reactive interval is carbonated, continued atmospheric CO_2_ removal requires the repeated production of fresh basaltic crust and the removal of carbonated crust from near-surface exchange. In other words, carbonated surface materials had to be transferred into the mantle or deep lithosphere, while fresh silicate rocks had to be continually supplied to the surface to sustain further weathering and carbonation [19,21,37]. Even under the generous assumption that Earth was entirely covered by magnesian oceanic crust (Table S1) and that the upper 500 m of globally distributed basaltic crust was completely weathered and carbonated, the Ca–Mg silicate inventory would sequester only on the order of ~47 bar of CO_2_, or perhaps ~52 bar if extensive Fe carbonation is included (Table S2). This capacity is substantially smaller than plausible post-magma-ocean CO_2_ inventories of ~100–200 bar, while continued mantle degassing would have replenished part of the atmospheric carbon removed by crustal carbonation. A hotter early mantle may have further increased this volcanic CO_2_ supply. Consequently, a single episode of shallow oceanic-crust alteration cannot by itself account for the large-magnitude CO_2_ drawdown required to move early Earth toward urable surface conditions (see Supplementary Information for detailed calculations).

This capacity argument points to the need for sustained crustal recycling on the early Earth. The key requirement was an early recycling mechanism capable of returning carbonated oceanic crust and sediments to depth while continually supplying fresh silicate rocks to the surface. Plate tectonics is the only known planetary-scale process that can sustain this coupled subduction–resurfacing cycle over geologic timescales, but in the Hadean context the term should be understood in a broad geodynamic sense: an efficient surface–interior recycling mechanism, rather than necessarily the full modern plate-boundary system characterized by long-lived rigid plates, organized spreading ridges, transform faults, and cold slab subduction. Such recycling would have helped lower *p*CO_2_, cool the surface, raise ocean pH, and maintain a relatively dry stratosphere, thereby reducing the risk of enhanced water loss through cold-trap failure. The importance of rapid early recycling has long been recognized in studies of early Earth surface environments and post-magma-ocean atmospheric evolution [17,18,19,21,64]. The possible operation of an early plate-tectonic or plate-like recycling regime is therefore central to the urability problem, because the same tectonic regime that regulated global CO_2_ removal also controlled continental growth, nutrient delivery, land emergence, and the local environments required by many prebiotic pathways.

A further consequence of sustained operation of plate tectonics is the generation and differentiation of continental crust. Recycling of hydrated and altered mafic crust can promote partial melting, arc magmatism, and intracrustal differentiation, producing more buoyant felsic to intermediate continental components that are compositionally distinct from basaltic oceanic crust. Because continental crust is enriched in incompatible elements, its growth would have progressively reorganized the chemical boundary conditions of Earth’s surface. This enrichment includes heat-producing elements such as thorium, uranium, and potassium, as well as bioessential and redox-sensitive elements including phosphorus and nitrogen, whose weathering and redistribution could have influenced nutrient availability, ocean chemistry, and local prebiotic reaction environments. Thus, early continental growth would have created new geochemical reservoirs and weathering substrates that fed back on climate regulation, surface redox structure, and the chemical diversity of habitable or prebiotic environments. Consistent with this view, an increasing number of studies based on igneous-rock geochemistry, isotopic mass balance, and noble-gas degassing point to substantial continental production during the Hadean, although the exact timing, volume, and preservation of this early crust remain debated [34,35,36,37,38,145,146,147].

**Figure 4:**
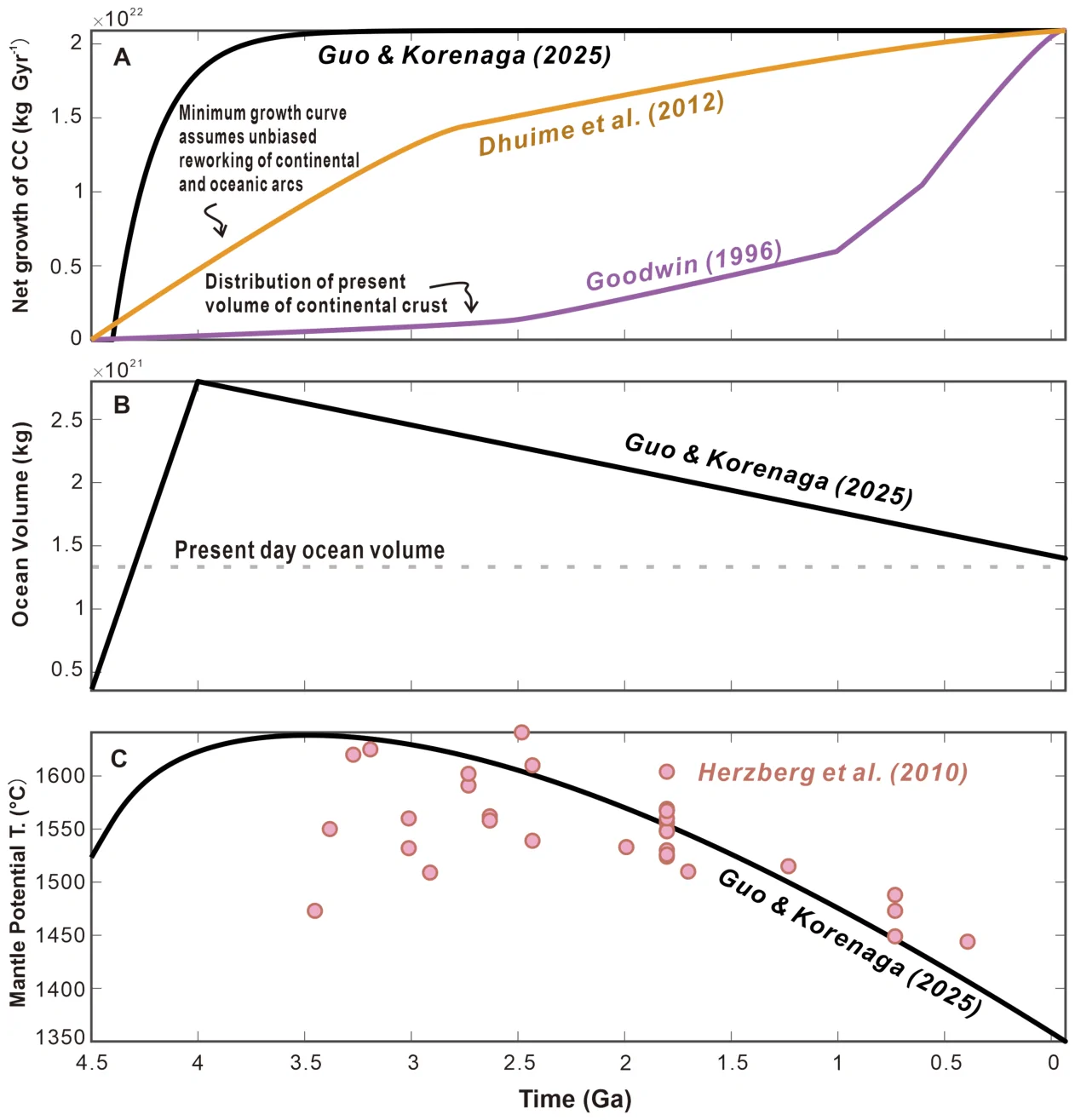
Long-term evolution of the continental crust, ocean, and mantle through Earth history. (**A**) Comparison of continental crust growth histories from different models and geological reconstructions [150]. The black line represents the rapid early-growth curve [35,36,37], the yellow curve shows the minimum growth scenario assuming unbiased reworking of continental crust and oceanic arcs [152], and the purple line denotes the estimated present-day continental crust volume [153]. (**B**) Modeled evolution of ocean volume over time [37], with the dashed horizontal line marking the present-day ocean volume. (**C**) Evolution of mantle potential temperature: the black curve shows the modeled cooling history [37], and circles represent independent petrological estimates [154].

The birth of early continental crust, however, was not equivalent to the continuous emergence of large subaerial landmasses. Continental exposure depends on freeboard, which reflects the balance among crustal thickness and buoyancy, ocean volume, seafloor depth, mantle temperature, ocean-basin capacity, and lithospheric strength. Thus, substantial continental crust could have remained largely submerged if early oceans were voluminous or if continents were thin, weak, and easily flooded; conversely, limited crustal volumes could still have generated exposed environments through buoyant blocks, volcanic islands, oceanic plateaus, or impact-generated topography [148,149,150,151]. Because continents, ocean volume, mantle temperature, and topography were all evolving during the Hadean–early Archean (Figure 4), land emergence was likely non-monotonic, with intervals of meaningful exposure alternating with periods dominated by ocean islands, transient shorelines, or submerged continental crust [38,149,150]. For prebiotic chemistry, the relevant variable is therefore not simply continental mass, but the time-dependent availability of exposed surfaces, coastlines, ephemeral basins, and shallow-water environments. Future work should quantify the coupled evolution of continental volume, freeboard, ocean volume, exposed land fraction, and shoreline length as boundary conditions for early Earth urability.

## 6. Conclusions

**Figure 5:**
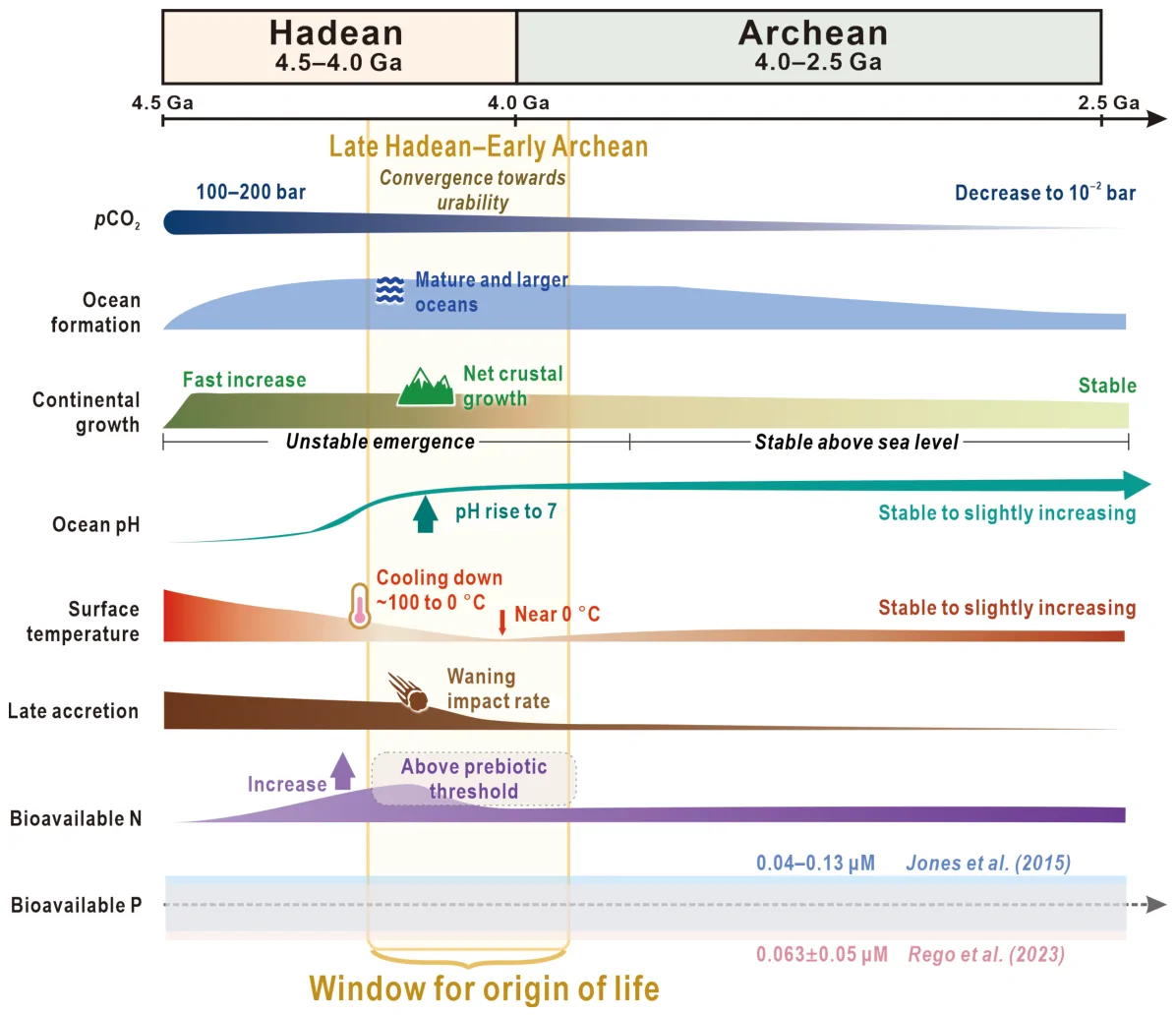
Multivariate evolution of early Earth surface conditions and the resulting window of opportunity for life’s origin. A convergence towards urability is reflected by the co-evolution of eight key parameters: decreasing atmospheric *p*CO_2_, ocean establishment and stabilization, net continental crustal growth, neutralization of ocean pH, surface cooling, declining impact rates, evolving nitrogen availability, and estimated phosphorus availability. Reconstructed trends draw upon atmospheric CO_2_ models [17,28,51,54,64], ocean formation, pH, and thermal histories [37], impact flux decay [39,155], crustal growth [35,36,37], nitrogen availability [33], and phosphorus availability [156,157]. The shaded zone highlights a conceptual window when multiple prebiotic prerequisites may have been simultaneously met, rather than representing a quantitatively constrained interval.

In summary, the origin-of-life problem should be framed not only as a question of chemical possibility, but also as a question of planetary timing. Early Earth likely passed through a sequence of rapidly changing states: ocean formation after magma-ocean solidification, drawdown of an initially CO_2_-rich atmosphere, moderation of surface temperature and ocean pH, changing nitrogen fixation and photochemical regimes, evolving ferruginous nutrient chemistry, and the progressive establishment of crustal recycling, continental growth, and intermittent land emergence (Figure 5). Each transition could have expanded some dimensions of prebiotic opportunity while restricting others. High *p*CO_2_ may have enhanced fixed-nitrogen production but imposed hot and acidic surface conditions; CO_2_ drawdown could have improved climate and ocean pH but reduced some abiotic NO_x_ sources; ferruginous chemistry could locally enhance phosphate availability while also promoting nutrient scavenging; and early tectonic recycling could stabilize the carbon cycle while creating heterogeneous land and shoreline environments. Urability therefore emerged from the transient overlap of these coupled constraints (Figure 5). Future progress will require moving beyond isolated origin-of-life settings toward quantitative Earth-system reconstructions that track when and where solvent availability, temperature, pH, nutrients, energy sources, atmospheric shielding, and exposed reactive surfaces jointly produced environments capable of sustaining prebiotic chemical evolution.

The broader planetary implication of this framework is that Earth’s path to urability was neither necessarily unique nor automatically reproducible. Some elements of the sequence described here—magma-ocean degassing, volatile redistribution, ocean condensation, silicate weathering, atmospheric evolution, and surface–interior exchange—should operate on many rocky planets with broadly Earth-like compositions. However, whether these processes generate urable environments depends on their timing and coupling. A planet with too much stellar irradiation, delayed CO_2_ drawdown, or inefficient volatile retention may move toward a Venus-like trajectory, whereas a planet with insufficient greenhouse warming, weak recycling, or strong atmospheric escape may fail to maintain persistent aqueous environments. Earth may therefore have been fortunate not because it possessed a single rare ingredient, but because multiple evolving conditions entered into overlap during the Hadean–early Archean transition. For other rocky planets, the same processes may proceed at different rates or fail to overlap, implying that urability should be evaluated as a probabilistic, time-dependent outcome of coupled planetary evolution rather than as a fixed property of location within the classical habitable zone.

## Data Availability

All calculations used to support the capacity arguments discussed in the manuscript are provided in the Supplementary Material. This review does not report new observational datasets.

## Supplementary Materials

The following supporting information can be downloaded at: https://www.mdpi.com/article/10.3390/life16091436/s1. Table S1. Oxide contents and density used for the carbonation-capacity calculation; Table S2. Formal carbonation capacity, full reaction of listed divalent-cation budget; Table S3. Sensitivity to reactive efficiency (high-Mg# crust, Ca + Mg, upper 500 m); Table S4. Conditional Fe-carbonation sensitivity (high-Mg# crust, upper 500 m).
