# Supplementary material for: The Emergence of an Urable Earth: How Early Planetary Evolution Shaped the Chemical Window for Life’s Origin
Source: Life (Basel). 2026 Aug 28;16(9):1436. doi: 10.3390/life16091436 (PMC13608292; doi:10.3390/life16091436)
Supplement: Supplementary file 1 [file life-16-01436-s001.zip › life-4433343-supplementary.pdf]

Supplementary Information:

The Emergence of an Urable Earth: How Early Planetary Evolution Shaped the Chemical Window for Life's Origin

Meng Guo [1,*], Zekun Meng [1,*], Siyu Liu [2] and Simon A. T. Redfern [3,4]

[1] GAIA Lab, Department of Earth and Planetary Sciences, The University of Hong Kong, Hong Kong 999077, China
[2] Department of Physics, Hong Kong University of Science and Technology, Hong Kong 999077, China
[3] Asian School of the Environment, Nanyang Technological University, 50 Nanyang Avenue, Singapore 639798, Singapore
[4] Earth Observatory of Singapore (EOS), Nanyang Technological University, 50 Nanyang Avenue, Singapore 639798, Singapore
* Correspondence: mengguo@hku.hk (M.G.); zkmeng@hku.hk (Z.M.)

Carbonation capacity of the upper Hadean oceanic crust

This calculation supports the capacity argument in Section 5 of the main text. The goal is to bracket how much atmospheric CO_2 could be consumed by one episode of carbonation in the upper oceanic crust, not to model the full kinetics of basalt alteration. We distinguish a formal stoichiometric ceiling, in which all Ca and Mg in a globally distributed 500 m layer react, from an accessibility-adjusted single-pass capacity, in which only part of the bulk Ca-Mg budget is effectively reached by fluids, dissolved, transported, and precipitated as carbonate. The latter is the basis for the approximately 20-40 bar Ca-Mg estimate quoted in the main text.

Important clarification. The reference Hadean oceanic crustal composition used here is the high-Mg# crust from Miyazaki and Korenaga (2022, Extended Data Table S2), with MgO = 21.5 wt%, CaO = 10.7 wt%, FeO = 5.3 wt%, and density = 3010 kg m^{-3}. Fe carbonation is retained only as a conditional upper-bound sensitivity because complete conversion of bulk FeO to carbonate depends on redox state, mineralogy, fluid chemistry, and kinetics.

1. Atmospheric CO_2 inventory per bar

The column mass of atmosphere supporting a surface partial pressure p is p/g, with $g = 9.81$ m s^{-2}. For $p = 1$ bar this is 1.02×10^4 kg m^{-2}. Multiplying by Earth's surface area $A_E = 5.10 \times 10^{14}$ m^2 and dividing by the molar mass of CO_2 (44.01 g mol^{-1}) gives:

$$N_{CO_2} \approx 1.18 \times 10^{20} \text{ mol } CO_2 \text{ per bar of surface partial pressure.}$$

2. Divalent-cation inventory and crustal compositions

Each mole of divalent cation (Ca^{2+}, Mg^{2+}, and where redox permits Fe^{2+}) sequesters one mole of CO_2 as carbonate. For a carbonated layer of thickness h over a wholly basaltic surface,

$$N_{div} = \rho\, A_E\, h \times \Sigma_i\, (w_i/M_i),$$

$$\text{capacity (bar)} = N_{div}/N_{CO_2}$$

where w_i and M_i are the mass fraction and molar mass of oxide i.

The reference composition is the high-Mg# crust from Miyazaki and Korenaga (2022, Extended Data Table 2). For comparison and sensitivity, the iron-rich crust and modern MORB compositions from the same table are also shown.

Table S1. Oxide contents and density used for the carbonation-capacity calculation.

Hadean Oceanic Crust Composition	SiO2	MgO	FeO	Al_2O_3	CaO	ρ (kg m^{-3})
High-Mg# crust	43.6	21.5	5.3	18.9	10.7	3010
Iron-rich crust	39.2	16	22.9	9.2	12.6	3570
Modern MORB	50.47	7.58	10.43	14.7	9.4	3030

3. Results: stoichiometric ceilings

Table S2 gives the formal stoichiometric capacity for complete conversion of the listed bulk CaO and MgO inventory into carbonates. Using the Miyazaki and Korenaga (2022) high-Mg# crust, full Ca-Mg carbonation of a global upper 500 m layer gives approximately 47 bar CO_2. If bulk FeO is also fully carbonated, the all-cation ceiling rises to approximately 52 bar, but this Fe-inclusive value should be regarded as a conditional endmember rather than the reference capacity.

Table S2. Formal carbonation capacity, full reaction of listed divalent-cation budget.

Composition and layer thickness	Ca + Mg	Ca + Mg + Fe
Modern MORB, upper 500 m	~23 bar	~33 bar
High-Mg# crust, upper 500 m	~47 bar	~52 bar
Iron-rich crust, upper 500 m	~48 bar	~72 bar

4. Accessibility-adjusted capacity used in the main text

The formal ceiling assumes whole-planet basaltic cover, a 500 m reactive interval everywhere, and complete utilization of the bulk Ca-Mg oxide budget. This is deliberately generous. In a single alteration episode, not all cations in the bulk rock need be equally accessible: reactive phases may be incompletely altered, fluids may not penetrate the entire interval uniformly, and carbonate precipitation may be limited by kinetics and transport. If 50-85% of the Ca-Mg budget is effectively accessible in the reference high-Mg# crust, the capacity is approximately 24-40 bar CO_2. This is the range reported in the main text as approximately 20-40 bar and is comparable in order of magnitude to the Hadean seafloor carbonate inventory estimated by Miyazaki and Korenaga (2022).

Table S3. Sensitivity to reactive efficiency (high-Mg# crust, Ca + Mg, upper 500 m).

Reactive fraction of Ca + Mg budget in the upper 500m crust	Capacity for CO_2 (in unit bar)
50%	~24 bar
70%	~33 bar
85%	~40 bar
100%	~47 bar

5. Conditional Fe-carbonation upper bound

Fe carbonation can increase the capacity only if Fe remains available as Fe^{2+} and if redox state, fluid composition, mineralogy, and kinetics permit substantial siderite or related Fe-carbonate formation. For this reason Fe is not included in the reference Ca-Mg estimate. As a conditional sensitivity test, adding complete

Fe carbonation to the upper part of the accessibility-adjusted Ca-Mg range gives approximately 45 bar CO_2. The absolute all-cation ceiling for the high-Mg# crust is approximately 52 bar and should be treated as a limiting endmember, not as the likely realized capacity.

Table S4. Conditional Fe-carbonation sensitivity (high-Mg# crust, upper 500 m).

Assumption	Capacity for CO_2 (in unit bar)
50% Ca+Mg plus complete Fe carbonation	~29 bar
70% Ca+Mg plus complete Fe carbonation	~38 bar
85% Ca+Mg plus complete Fe carbonation	~45 bar
100% Ca+Mg plus complete Fe carbonation	~52 bar

6. Implication

These calculations reconcile the main-text values with the stoichiometric calculation. For the Miyazaki and Korenaga (2022) high-Mg# crust, the formal Ca-Mg stoichiometric ceiling for a globally distributed 500 m layer is approximately 47 bar. However, an accessibility-adjusted single-pass capacity is approximately 20-40 bar, and extensive Fe carbonation could raise the upper end to approximately 45 bar under favorable redox, mineralogical, and kinetic conditions. In all cases, the capacity remains substantially smaller than plausible post-magma-ocean CO_2 inventories of approximately 100-200 bar, especially if continued mantle degassing replenished part of the atmospheric carbon removed by crustal carbonation. Therefore, a single episode of shallow oceanic-crust alteration cannot by itself account for the required large-magnitude CO_2 drawdown. Sustained drawdown requires both removal of carbonated crust from near-surface exchange and continual exposure of fresh silicate rocks, implying an efficient surface-interior recycling mechanism operating in a broad geodynamic sense.

Caveats. First, the carbonated interval is limited by fluid access; thicker Hadean crust does not automatically increase capacity if alteration is concentrated in the upper few hundred metres. Second, Mg-bearing silicate carbonation may be kinetically limited, especially at low temperature, so complete use of the Mg budget is unlikely in all settings. Third, Fe carbonation is redox- and mineralogy-dependent and is treated only as a conditional upper bound. Fourth, the calculation uses bulk oxide contents rather than modal abundances of reactive phases, so it is intended as an order-of-magnitude capacity bracket, not a kinetic alteration model.

Supplementary Reference

Miyazaki, Y.; Korenaga, J. A wet heterogeneous mantle creates a habitable world in the Hadean. *Nature* 2022, *603*, 86–90. https://doi.org/10.1038/s41586-021-04371-9.
